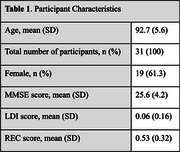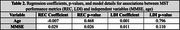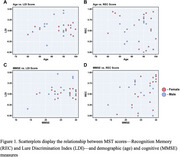# Mnemonic Similarity Task Reveals Feasibility and Insights into Memory Performance in Older Adults

**DOI:** 10.1002/alz70857_107175

**Published:** 2025-12-26

**Authors:** Ghasem Farahmand, Katelynn M. Nguyen, Sam T Gouron, Joshua K Cheung, Craig EL Stark, Seyed Ahmad Sajjadi

**Affiliations:** ^1^ University of California, Irvine, Irvine, CA, USA; ^2^ Department of Neurobiology and Behavior, University of California, Irvine, Irvine, CA, USA

## Abstract

**Background:**

The Mnemonic Similarity Task (MST) is a behavioral tool designed to tax pattern separation, a critical hippocampal function involving the transformation of similar experiences into discrete, non‐overlapping memory representations. Our pilot study aimed to assess the feasibility of MST administration among the oldest old, individuals 80 years and older, explore the association of MST performance metrics with established global cognitive measures, and evaluate potential age effects.

**Methods:**

Thirty‐one participants aged 80 years and older from the UCI Alzheimer's Disease Research Center (ADRC) and the 90+ Study underwent MST as part of their cognitive assessment. Participants categorized 64 images as “indoor” or “outdoor” during the encoding phase and later classified 96 images as “old,” “similar,” or “new” in a recognition phase. Images were categorized as targets (old), lures (similar), or foils (novel). Key outcome measures included recognition memory (REC) and the lure discrimination index (LDI). Linear regression analyses were conducted to evaluate the associations between MST scores, age, and cognitive measures, i.e. the Mini‐Mental State Examination (MMSE).

**Results:**

Mean age was 92.7± 5.6 and 61.3% were female (Table 1). MST was successfully completed by all participants. Regression analyses revealed no significant association between MST scores and age (REC: *p* =  0.12; LDI: *p* =  0.09) (Table 2). A significant positive correlation was identified between MMSE and REC scores (β = 0.36, *p* =  0.02). No significant association was found between MMSE and LDI (β = 0.15, *p* =  0.28). Scatterplots indicated variability in individual MST scores with no discernible age‐related decline. (Figure 1)

**Conclusion:**

The MST is a feasible and effective task for assessing pattern separation and recognition memory in the oldest old. The observed association between MMSE and REC, but not LDI, deserves further investigation. The direct relationship between REC and MMSE underscores the utility of MST as a cognitive assessment tool. Further research with expanded cognitive measures and imaging biomarkers is warranted to elucidate the underlying mechanisms driving MST performance and its role as a digital biomarker for cognitive health.